# Acute paraxanthine ingestion increases energy, focus, and satiety, enhancing postprandial cognitive performance in young adults

**DOI:** 10.1080/15502783.2025.2550171

**Published:** 2025-12-23

**Authors:** Sidney Abou Sawan, Ralf Jäger, Raza Bashir

**Affiliations:** a Scientific Affairs, Iovate Health Sciences International Inc, Oakville, ON, Canada; b Increnovo LLC, Whitefish Bay, WI, USA; c Ingenious Ingredients L.P, Lewisville, TX, USA

**Keywords:** Supplement, cognition, productivity, appetite

## Abstract

**Background:**

Paraxanthine, when ingested in doses of 50–200 mg in the fasted state, enhances cognitive performance, including memory, reaction time, and attention, while suppressing hunger. However, unlike caffeine, paraxanthines effects on energy, focus, appetite, and cognition in the fed state are unexplored. Our objective was to determine if paraxanthine can increase energy, focus, cognitive performance, and manage appetite in young healthy adults in the fed state.

**Methods:**

We conducted a double-blind, randomized, placebo-controlled, crossover study with 24 young, healthy adults (12 males, 12 females; age: 24 ± 5 years; BMI: 24.7 ± 2.3 kg/m^2^). Participants consumed MuscleTech® paraxanthine at 200 mg (PXN 200), 300 mg (PXN 300), or placebo (PLA) after an overnight fast and 30 minutes before a mixed meal (340 kcal: 46 g carbs, 29.5 g protein, 6 g fat). Measures of energy, focus, productivity, satiety, and appetite were assessed using a visual analogue scale, along with cognitive performance tests (N-Back, Go/No-Go, Serial 7s), were taken at −30 minutes (pre-product ingestion), at 0 (pre-meal ingestion), and 30-, 60-, 120-, and 180-minutes post-meal.

**Results:**

Both doses of paraxanthine increased energy and productivity compared to PLA (*p* < 0.01), with PXN 200 showing superior effects over PXN 300 (*p* < 0.05). PXN 200 also increased focus and satiety more than PLA (*p* < 0.005) and showed a trend over PXN 300 (*p* = 0.052), without affecting appetite (*p* = 0.53). In cognitive tasks, PXN 200 and PXN 300 improved performance on the N-Back task by increasing engagement (*p* < 0.01) and correct responses (*p* < 0.05) compared to PLA. PXN 300 specifically reduced time per score compared to PLA (*p* < 0.05) and increased overall score compared to PLA (*p* < 0.01), while PXN 200 showed temporary enhancements at 60 and 120 minutes compared to −30 minutes (*p* < 0.01). On the Go/No-Go task, PXN 300 improved Go responses compared to PLA (*p* < 0.05) with a trend toward better performance than PXN 200 (*p* = 0.056), while PXN 200 improved reaction time at 120 minutes compared to −30 minutes (*p* < 0.05). No effects were observed in No-Go performance (accuracy or reaction time) or Serial 7s compared to PLA (*p* ≥ 0.26), indicating paraxanthine does not influence impulse control or arithmetic ability in the fed state.

**Conclusions:**

Our data suggest that paraxanthine, particularly at the 200 mg dose, can enhance subjective feelings of energy, focus, productivity, and satiety, as well as cognitive improvements in attention and processing speed in the fed state.

